# Cancer Risk Associated with Insulin Glargine among Adult Type 2 Diabetes Patients – A Nationwide Cohort Study

**DOI:** 10.1371/journal.pone.0021368

**Published:** 2011-06-27

**Authors:** Chia-Hsuin Chang, Sengwee Toh, Jou-Wei Lin, Shu-Ting Chen, Chuei-Wen Kuo, Lee-Ming Chuang, Mei-Shu Lai

**Affiliations:** 1 Institute of Preventive Medicine, College of Public Health, National Taiwan University, Taipei, Taiwan; 2 Department of Internal Medicine, National Taiwan University Hospital, Taipei, Taiwan; 3 Department of Population Medicine, Harvard Medical School/Harvard Pilgrim Health Care Institute, Boston, Massachusetts, United States of America; 4 Cardiovascular Center, National Taiwan University Hospital Yun-Lin Branch, Dou-Liou City, Yun-Lin, Taiwan; 5 National Health Insurance Mediation Committee, Department of Health, Executive Yuan, Taipei, Taiwan; University of Tor Vergata, Italy

## Abstract

**Background:**

Preclinical and observational studies raise the concern about the safety of insulin glargine in terms of cancer initiation and promotion. This study is designed to examine cancer incidence associated with use of insulin glargine vs. intermediate/long-acting human insulin (HI).

**Methodology:**

A retrospective cohort study using the Taiwan National Health Insurance claims database was conducted to identify adult patients with type 2 diabetes mellitus and without a history of cancer who initiated insulin glargine (n = 10,190) or intermediate/long-acting HI (n = 49,253) during 2004–2007. Exclusive users were followed from the date of insulin initiation to the earliest of cancer diagnosis, death, disenrollment, or December 31 2007. We estimated adjusted hazard ratios and 95% confidence intervals (CIs) with Cox proportional hazards models adjusting for baseline propensity score.

**Findings:**

The incidence rate of all cancer per 1,000 person-years was 13.8 for insulin glargine initiators (179 cases) and 16.0 for intermediate/long-acting HI initiators (1,445 cases) during an average follow-up of 2 years. No significant difference in overall cancer risk between insulin glargine initiators and HI initiators was found. For men, however, the adjusted hazard ratio of insulin glargine use as compared with intermediate/long-acting HI was 2.15 (95% CI 1.01–4.59) for pancreatic cancer, and 2.42 (95% CI 1.50–8.40) for prostate cancer. The increased risk was not observed among women.

**Conclusions:**

Insulin glargine use did not increase the risk of overall cancer incidence as compared with HI. The positive associations with pancreatic and prostate cancer need further evaluation and validation.

## Introduction

During the past decades, long-acting insulin preparations have become widely used as a basal insulin supplement for diabetic patients due to their stable action and lower risk of nocturnal hypoglycemia. However, modification of amino acids on the insulin chain for these new insulin analogues may not only change metabolic properties but also alter their mitogenic effects, probably through prolonged binding to insulin receptor or by increased cross-reactivity with IGF-1 receptor [1]. Several studies showed that as compared with human insulin, insulin glargine – a long-acting insulin analogue – might substantially increase cellular proliferative potential, while the mitogenic potency of the other insulin analogues, including insulin detemir, were similar to or lower than human insulin [2]. In addition, it has been shown that insulin glargine, but not human insulin, increases resistance to apoptosis in several tumor cell lines including colorectal, breast, and prostate cancers [3]. These preclinical studies raise the concern about a potential link between insulin glargine and cancer initiation and promotion.

While one open-label randomized trial and a combined analysis of 31 randomized controlled trials (mostly of 6-month duration) found no difference in cancer occurrence between insulin glargine and comparative groups (mostly neutral protamine Hagedorn insulin) [4], [5], observational studies analyzing large electronic healthcare databases or diabetes registry showed conflicting results [6]–[10]. A cohort study from Germany reported that the risk of overall cancer increased with dose for any type of insulin. The hazard ratio for overall insulin glargine use as compared with human insulin was 0.86. However, at doses greater than 40 IU, users of insulin glargine but not insulin aspart and insulin lispro had a higher risk than individuals using human insulin [6]. Similar findings were observed in a nested case-control study from Italy showing that the incidence of overall cancer was associated with a daily dose of insulin glargine ≥0.3 IU/kg but not for human insulin or other analogues [7]. Additionally, two studies in Sweden and Scotland suggested that women using insulin glargine alone had a significantly higher risk of breast cancer as compared with users of other types of insulin, whereas this increased risk was not observed among those who received insulin glargine in combination with other insulin [8], [9]. In contrast, a UK study found that despite a higher risk of overall cancer among diabetic patients receiving insulin or sulfonylurea compared to those using metformin, the risk was similar for different insulin formulations at the doses used in clinical practice [10].

Residual confounding, reverse causation, selection or detection biases might have influenced the validity of these studies [11], [12]. In this study, we examined whether cancer incidence was associated with the use of insulin glargine compared to intermediate/long-acting human insulin (HI) using the Taiwan National Health Insurance claims database.

## Results

**Figure 1:**
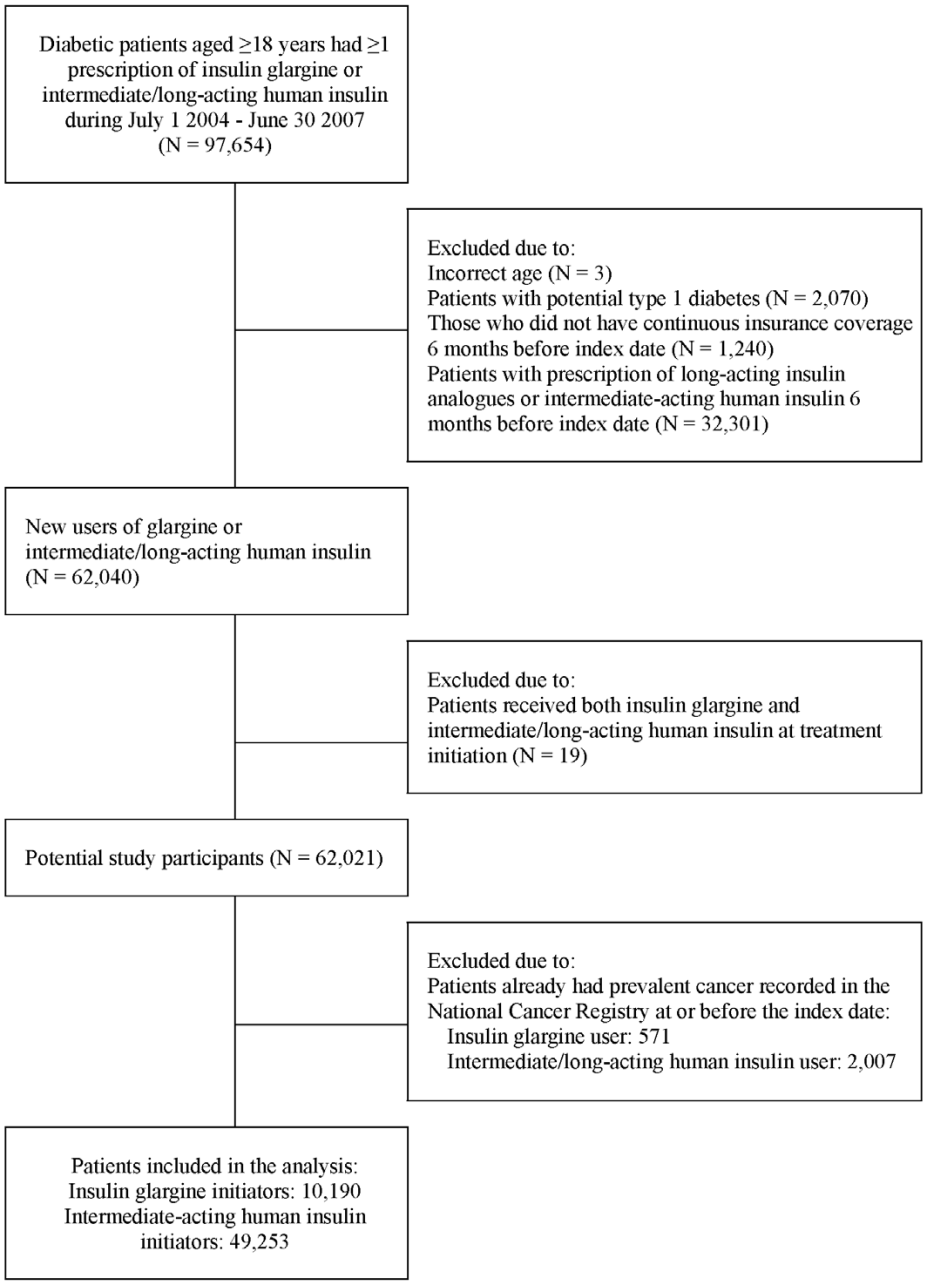
Study flow.

A total of 10,190 insulin glargine initiators and 49,253 intermediate/long-acting HI initiators were included in the analysis (Figure 1). These two treatment groups differed in many baseline characteristics (Table 1). As compared with intermediate/long-acting HI initiators, those who starting insulin glargine therapy were more likely to have history of ketoacidosis or non-ketotic hyperosmolarity and retinopathy, but less likely to have cerebrovascular and peripheral vascular diseases, nephropathy, chronic kidney and lung disease; were mostly cared for by endocrinologists and in the medical centers. A significantly higher proportion of insulin glargine initiators also received oral anti-diabetic agents and statins, while less received fast-acting insulin therapy during the 6-month period prior to the initiation of insulin glargine. Insulin glargine initiators received more frequent hemoglobin A_1_C measurements and have more frequent outpatient visits due to diabetes but less likely to be hospitalized due to either diabetes or non-diabetes problems.

**Table 1:** Baseline characteristics during the 6-month period before initiation of insulin glargine or intermediate/long-acting human insulin (HI).

	Insulin glargine initiators(N = 10,190)	HI initiators(N = 49,253)	Standardized mean difference/Odds ratio (95% CI)
*Patient characteristics*			
Age at studied insulin initiation (mean ± SD)	60.65±12.84	62.07±13.41	−0.08
≤34	2.5	3.0	Reference
35–44	8.3	7.3	1.38 (1.18, 1.60)
45–54	21.2	18.4	1.38 (1.20, 1.59)
55–64	27.7	25.2	1.32 (1.15, 1.52)
≥65	40.3	46.1	1.05 (0.92, 1.21)
Male (%)	49.5	47.7	1.08 (1.03, 1.12)
Initiation year (%)			
2004	7.3	32.6	Reference
2005	20.9	31.8	2.94 (2.70, 3.20)
2006	41.8	25.1	7.43 (6.85, 8.06)
2007	30.0	10.6	12.61 (11.57, 13.75)
Diabetes-related late complications (%)			
Cardiovascular disease	66.9	66.4	1.02 (0.97, 1.07)
Ischemic heart disease	19.2	20.3	0.93 (0.89, 0.99)
Peripheral vascular disease	2.2	2.8	0.79 (0.69, 0.91)
Cerebrovascular disease	12.6	17.1	0.70 (0.66, 0.74)
Ketoacidosis or hyperosmolarity	24.6	17.8	1.51 (1.43, 1.59)
Retinopathy	33.2	30.3	1.14 (1.09, 1.20)
Neuropathy	22.7	21.7	1.06 (1.01, 1.11)
Nephropathy	36.5	40.6	0.84 (0.81, 0.88)
Chronic kidney disease	8.1	11.1	0.71 (0.66, 0.76)
Chronic liver disease	13.5	13.5	1.00 (0.94, 1.07)
Chronic lung disease	9.1	14.1	0.61 (0.57, 0.66)
Depression	2.7	2.8	0.94 (0.82, 1.07)
Charlson's index (not include diabetes) ≥1	47.2	54.5	0.75 (0.72, 0.78)
*Medication use (%)*			
Biguanides	76.0	62.1	1.93 (1.83, 2.02)
Sulfonylurea	79.3	67.8	1.82 (1.73, 1.92)
Alpha-glucosidase inhibitors	36.6	22.5	1.99 (1.91, 2.09)
Thiazolidinediones	43.5	25.9	2.21 (2.11, 2.31)
Glinides	17.4	14.4	1.26 (1.19, 1.33)
Any oral anti-diabetic agents	92.0	79.2	3.03 (2.81, 3.26)
Fast-acting insulins	28.5	42.0	0.55 (0.53, 0.58)
Premixed insulin	20.8	19.7	1.07 (1.02, 1.13)
Insulin detemir	1.4	0.2	6.40 (4.97, 8.25)
Statins	36.3	27.1	1.54 (1.47, 1.61)
Aspirin	34.4	33.2	1.06 (1.01, 1.11)
*Resource utilization (%))*			
Number of A_1_C measurement	10.9±6.1	8.7±5.9	0.03
0	16.4	36.0	Reference
1–2	31.7	29.4	2.36 (2.22, 2.51)
3–4	32.6	23.4	3.06 (2.87, 3.26)
>4	19.2	11.2	3.76 (3.49, 4.04)
Number of outpatient visits due to diabetes	8.3±4.7	7.4±5.2	−0.10
0–5	34.9	43.2	Reference
6–10	47.4	40.6	1.45 (1.38, 1.52)
>10	17.7	16.2	1.35 (1.27, 1.43)
Number of outpatient visits not due to diabetes			
0–10	61.6	55.4	Reference
11–20	23.9	26.5	0.81 (0.77, 0.85)
>20	14.6	18.1	0.73 (0.68, 0.77)
Emergency department visit	27.0	32.2	0.78 (0.74, 0.82)
Gastrointestinal specialist	12.9	13.3	0.96 (0.90, 1.03)
Urology specialist	8.0	8.0	1.01 (0.93, 1.09)
Chest medicine physicians	6.1	7.4	0.81 (0.74, 0.88)
Abdominal sonographic examination	11.7	11.9	0.99 (0.92, 1.05)
Colonoscopic examinations	0.1	0.1	0.86 (0.45, 1.63)
Mammographic examinations	0.8	0.6	1.34 (1.05, 1.72)
Serum prostate specific antigen measurement	1.9	1.6	1.16 (0.99, 1.36)
Chest x ray examinations	26.7	32.2	0.77 (0.73, 0.80)
Hospitalization due to diabetes	19.1	23.5	0.77 (0.73, 0.81)
Hospitalization not due to diabetes	15.0	23.2	0.58 (0.55, 0.62)
*Physician characteristics*			
Age (mean ± SD)	48.83±15.54	47.72±13.07	0.10
≤34	13.1	10.0	Reference
35–44	38.2	40.7	0.72 (0.67, 0.77)
45–54	23.5	28.8	0.62 (0.58, 0.67)
55–64	8.3	7.9	0.79 (0.72, 0.87)
≥65	17.0	12.6	1.03 (0.95, 1.12)
Male (%)	82.1	88.8	0.58 (0.55, 0.61)
Specialty (%)			
Endocrinologist	60.2	38.0	2.43 (2.28, 2.58)
General internist	25.6	40.4	0.97 (0.90, 1.04)
Family doctor and others	14.2	21.7	Reference

SD: Standard deviation.

### “Exclusive users” analysis

The average follow-up duration for insulin glargine initiators was 526 days, shorter than 745 days for intermediate/long-acting HI initiators (Table 2). Insulin glargine initiators were more likely to use oral anti-diabetic agents, metformin, and sulfonylurea whereas intermediate/long-acting HI initiators more often used fast-acting insulins as concomitant medications. The mean daily dosage of studied insulin was 0.48 DDD (19.2 IU/day) for glargine users as compared with 0.41 DDD (16.4 IU/day) for intermediate/long-acting HI users. In addition, the mean daily dosage for all anti-diabetic agents, including sulfonylurea (1.26 *vs*. 0.98 DDD) and metformin (0.59 *vs.* 0.47 DDD) among users were higher in insulin glargine initiators.

**Table 2:** Follow-up in days and medication use in exclusive users of insulin glargine compared to intermediate/long-acting human insulin (HI).

	Insulin glargine initiators(N = 9,041)	HI initiators(N = 44,274)
Follow-up days (mean ± SD)	525.90±284.57	745.36±366.84
*Medication use*		
Fast-acting insulins	1827 (20.2%)	22803 (51.5%)
Insulin detemir	677 (7.5%)	1883 (4.3%)
Any oral anti-diabetic medications	8349 (92.3%)	34291 (77.5%)
Sulfonylurea	6597 (73.0%)	27604 (62.3%)
Metformin	6310 (69.8%)	27034 (61.1%)
*Daily dosage in DDD among users (mean ± SD)*		
Studied insulin	0.48±0.48	0.41±0.65
Fast-acting insulin	0.30±0.53	0.30±0.85
Insulin detemir	0.19±0.18	0.14±0.16
Any oral anti-diabetic medications	1.81±1.28	1.40±1.21
Sulfonylurea	1.26±1.01	0.98±0.87
Metformin	0.59±0.38	0.47±0.37

SD: Standard deviation.

A total of 179 cancer cases in the insulin glargine initiators and 1,445 cancer cases in the intermediate/long-acting HI initiators occurred during follow-up. The crude incidence rate of all cancers per 1,000 person-year was 13.8 (95% CI: 11.7–15.8) for insulin glargine initiators and 16.0 (95% CI: 15.2–16.8) for intermediate/long-acting HI initiators (Table 3). No significantly increased risk of cancer was found for insulin glargine initiators as compared with intermediate/long-acting HI initiators. The crude hazard ratio for any cancer was 0.84 (95% CI: 0.72–0.98); the hazard ratio was 0.86 (95% CI: 0.72–1.01) after adjusting for baseline propensity score. Results were similar in the traditional multivariable-adjusted model and extended Cox model controlling for baseline propensity score, time-varying anti-diabetic medication use, and mean daily dosage of studied insulin during the study period (Table 4). For individual sites of cancer, a significantly increased risk was found for pancreatic cancer, particularly in men. The adjusted hazard ratio was 2.15 (95% CI 1.01–4.59) for pancreatic cancer, and 2.42 (95% CI 1.50–8.40) for prostate cancer (Table 4 and Table 5). The increased risk was not observed among women (Table 5).

**Table 3:** Crude incidence rate of any cancer and individual site of cancer among exclusive users of insulin glargine *vs.* intermediate/long-acting human insulin (HI).

	Insulin glargine initiators(Total follow-up 13017.6 person-years)		HI initiators(Total follow-up 90349.0 person-years)	
	Number of cases	Incidence rate per 1,000 person-years(95% confidence interval)	Number of cases	Incidence rate per 1,000 person-years(95% confidence interval)
Any cancer	179	13.8 (11.7, 15.8)	1445	16.0 (15.2, 16.8)
Breast^a^	6	0.9 (0.2, 1.6)	65	1.4 (1.0, 1.7)
Colorectal	23	1.8 (1.0, 2.5)	205	2.3 (2.0, 2.6)
Stomach	7	0.5 (0.1, 0.9)	79	0.9 (0.7, 1.1)
Pancreas	23	1.8 (1.0, 2.5)	73	0.8 (0.6, 1.0)
Liver	42	3.2 (2.3, 4.2)	405	4.5 (4.0, 4.9)
Lung	19	1.5 (0.8, 2.1)	137	1.5 (1.3, 1.8)
Prostate^b^	9	1.4 (0.5, 2.3)	29	0.7 (0.4, 0.9)
Kidney and urinary bladder	8	0.6 (0.2, 1.0)	98	1.1 (0.9, 1.3)
Skin	8	0.6 (0.2, 1.0)	54	0.6 (0.4, 0.8)

a Total follow-up person-year for women: 6,558.8 for insulin glargine users and 47,724.6 for HI users.

b Total follow-up person-year for men: 6,458.8 for insulin glargine users and 42,624.4 for HI users.

**Table 4:** Hazard ratio of overall and individual cancer among exclusive users of insulin glargine vs. intermediate/long-acting human insulin (HI) users.

	Unadjusted	Traditional multivariable adjusted^a^	Adjusted for baseline propensity score	Adjusted for baseline propensity score and time-varying medication use^b^	Adjusted for baseline propensity score, time-varying medication use and dosage of studied insulin
Any cancer	0.84(0.72, 0.98)	0.86(0.73, 1.01)	0.86(0.72, 1.01)	0.85(0.72, 1.01)	0.86(0.73, 1.02)
Breast (women)	0.71(0.31, 1.65)	0.62(0.25, 1.51)	0.56(0.23, 1.36)	0.55(0.22, 1.35)	0.53(0.21, 1.31)
Colorectal	0.79(0.51, 1.22)	0.86(0.54, 1.36)	0.85(0.53, 1.35)	0.80(0.5, 1.28)	0.78(0.49, 1.26)
Stomach	0.60(0.27, 1.30)	0.65(0.28, 1.47)	0.59(0.26, 1.35)	0.63(0.27, 1.44)	0.62(0.27, 1.42)
Pancreas	1.89(1.17, 3.03)	1.69(0.99, 2.87)	1.83(1.07, 3.12)	1.88(1.08, 3.27)	1.85(1.06, 3.22)
Liver	0.70(0.51, 0.97)	0.74(0.53, 1.04)	0.74(0.53, 1.04)	0.74(0.52, 1.04)	0.76(0.54, 1.08)
Lung	0.97(0.60, 1.57)	0.97(0.58, 1.63)	0.98(0.58, 1.64)	0.98(0.58, 1.66)	1.01(0.59, 1.71)
Prostate (men)	2.01(0.94, 4.29)	2.27(0.94, 5.48)	2.42(1.50, 8.40)	2.42(0.96, 6.09)	2.37(0.94, 6.01)
Kidney and urinary bladder	0.54(0.26, 1.12)	0.59(0.27, 1.26)	0.60(0.28, 1.29)	0.53(0.25, 1.15)	0.54(0.25, 1.16)
Skin	1.06(0.50, 2.24)	1.14(0.51, 2.57)	1.18(0.53, 2.64)	1.17(0.52, 2.64)	1.08(0.48, 2.46)

a Adjusted for all variables in Table 1.

b Time-varying medication use included insulin detemir (binary), mean daily dosage of fast-acting insulins, sulfonylurea, and metformin (in quartiles).

**Table 5:** Hazard ratio of overall and individual cancer comparing exclusive use of insulin glargine with intermediate/long-acting human insulin (HI) among men and women.

	Women		Men	
	Unadjusted	Adjusted for baseline propensity score	Unadjusted	Adjusted for baseline propensity score
Any cancer	0.78(0.61, 1.00)	0.71(0.55, 0.92)	0.87(0.71, 1.07)	0.97(0.78, 1.22)
Colorectal	0.89(0.49, 1.63)	0.78(0.41, 1.51)	0.70(0.37, 1.30)	0.92(0.47, 1.80)
Stomach	0.75(0.23, 2.47)	0.64(0.18, 2.31)	0.50(0.18, 1.40)	0.59(0.20, 1.72)
Pancreas	2.00(1.01, 3.96)	1.43(0.66, 3.11)	1.78(0.92, 3.42)	2.15(1.01, 4.59)
Liver	0.56(0.32, 0.98)	0.53(0.29, 0.96)	0.78(0.53, 1.15)	0.84(0.55, 1.29)
Lung	0.87(0.37, 2.05)	0.84(0.33, 2.12)	0.99(0.55, 1.79)	1.03(0.55, 1.95)
Kidney and urinary bladder	0.53(0.19, 1.48)	0.64(0.22, 1.91)	0.55(0.20, 1.53)	0.48(0.16, 1.42)
Skin	0.71(0.22, 2.34)	0.71(0.20, 2 .55)	1.52(0.57, 4.08)	1.74(0.60, 5.07)

In the analyses stratified on different dose and duration categories, we found no significant difference in the risk of overall cancer between insulin glargine initiators and HI initiators, among those with higher cumulative dose (>300 DDD), higher mean daily dosage (≥0.5 DDD/day) or longer cumulative treatment duration (≥1 years) (Table 6). Relatively few patients had pancreatic and prostate cancer in each dose and duration categories and the confidence intervals were wide. However, insulin glargine was potentially associated with a significantly increased risk of pancreatic cancer with cumulative dosage ≥300 DDD; the adjusted hazard ratio was 7.75 (95% CI: 2.64–22.72). No significantly excess risk was found for prostate cancer in different dose- and duration categories.

**Table 6:** Hazard ratio of overall cancer and pancreatic cancer among exclusive users of insulin glargine vs. intermediate/long-acting human insulin (HI) users.

	Hazard Ratio (95% CI)		
	Crude	Adjusted for baseline propensity score^a^	Adjusted for baseline propensity score and time-varying medication use^b^
Overall cancer			
Cumulative dosage			
>300 DDD	1.05 (0.77, 1.43)	1.17 (0.83, 1.64)	1.18 (0.83, 1.67)
50–135 DDD	0.70 (0.49, 0.98)	0.77 (0.53, 1.12)	0.79 (0.54, 1.16)
135–300 DDD	0.69 (0.48, 0.98)	0.74 (0.50, 1.09)	0.75 (0.50, 1.11)
<50 DDD	0.90 (0.69, 1.18)	0.82 (0.61, 1.09)	0.81 (0.6, 1.07)
Cumulative duration			
≥1 years	0.86 (0.60, 1.23)	0.88 (0.59, 1.31)	0.91 (0.61, 1.36)
<1 years	0.82 (0.69, 0.98)	0.83 (0.69, 1.01)	0.83 (0.69, 1.01)
Mean daily dosage			
≥0.5 DDD/day	1.04 (0.84, 1.28)	1.06 (0.85, 1.33)	1.05 (0.84, 1.32)
<0.5 DDD/day	0.65 (0.51, 0.83)	0.65 (0.50, 0.85)	0.68 (0.52, 0.89)
Pancreatic cancer			
Cumulative dosage			
>300 DDD	4.28 (1.68, 10.88)	7.75 (2.64, 22.72)	7.90 (2.64, 23.71)
135–300 DDD	0.97 (0.28, 3.40)	1.12 (0.26, 4.80)	1.15 (0.27, 5.00)
50–135 DDD	1.11 (0.37, 3.34)	1.27 (0.37, 4.36)	1.19 (0.34, 4.17)
<50 DDD	2.05 (0.96, 4.38)	1.18 (0.51, 2.72)	1.23 (0.53, 2.84)
Cumulative duration			
≥1 years	1.27 (0.28, 5.71)	2.28 (0.44, 11.78)	2.58 (0.50, 13.23)
<1 years	1.94 (1.17, 3.19)	1.70 (0.96, 3.01)	1.75 (0.98, 3.12)
Mean daily dosage			
≥0.5 DDD/day	2.20 (1.12, 4.33)	1.85 (0.87, 3.94)	1.84 (0.86, 3.95)
<0.5 DDD/day	1.61 (0.83, 3.12)	1.78 (0.81, 3.94)	1.92 (0.85, 4.32)
Prostate cancer (men)			
Cumulative dosage			
>300 DDD	2.20 (0.45, 10.72)	1.73 (0.28, 10.68)	2.16 (0.33, 14.10)
135–300 DDD	3.07 (0.68, 13.80)	5.28 (0.73, 37.93)	5.37 (0.71, 40.85)
50–135 DDD	2.31 (0.41, 12.94)	1.69 (0.26, 10.88)	2.06 (0.29, 14.7)
<50 DDD	1.07 (0.24, 4.87)	2.03 (0.33, 12.38)	1.88 (0.30, 11.63)
Cumulative dosage			
≥180 DDD	2.66 (0.80, 8.82)	2.02 (0.47, 8.71)	2.39 (0.53, 10.76)
<180 DDD	1.55 (0.57, 4.20)	2.46 (0.77, 7.92)	2.38 (0.73, 7.78)
Cumulative duration			
≥1 years	1.85 (0.37, 9.18)	1.65 (0.26, 10.44)	1.79 (0.26, 12.33)
<1 years	1.93 (0.81, 4.58)	2.61 (0.92, 7.44)	2.52 (0.87, 7.25)
Mean daily dosage			
≥0.5 DDD/day	1.55 (0.44, 5.41)	1.91 (0.46, 7.91)	1.98 (0.47, 8.40)
<0.5 DDD/day	2.21 (0.83, 5.87)	2.83 (0.84, 9.54)	2.95 (0.83, 10.43)

a Extended Cox model with time-varying insulin glargine and HI use, controlling for baseline propensity score.

b Extended Cox model with time-varying insulin glargine and HI use, controlling for baseline propensity score and time-varying use of insulin detemir (binary), mean daily dosage of fast-acting insulins, sulfonylurea, and metformin (in quartiles).

### “As-treated” analysis

In this analysis, we followed all studied insulin users to the earliest of cancer diagnosis, death, disenrollment, discontinuing studied insulin or switching to another insulin, or study end. The average follow-up duration was 238 days for insulin glargine initiators and 205 days for intermediate/long-acting HI initiators.

A total of 78 cancer cases in the insulin glargine initiators and 390 cancer cases in the intermediate/long-acting HI initiators occurred during follow-up. The crude incidence rate of all cancers per 1,000 person-year was 11.7 (95% CI: 9.1–14.3) for insulin glargine initiators and 14.1 (95% CI: 12.7–15.5) for intermediate/long-acting HI initiators. No significantly increased risk of cancer was found for insulin glargine initiators as compared with intermediate/long-acting HI initiators. The crude hazard ratio for any cancer was 0.83 (95% CI: 0.65–1.06); the hazard ratio was 0.81 (95% CI: 0.62–1.05) after adjusting for baseline propensity score. Similarly, a significantly increased risk was found among men with the adjusted hazard ratio of 3.38 (95% CI 1.18–9.66) for pancreatic cancer, and 4.44 (95% CI 1.12–17.68) for prostate cancer (Supplementary Tables S2 and S3).

In the analyses stratified on different dose and duration categories, we found no significant difference in the risk of overall cancer between insulin glargine initiators and HI initiators, although relatively few patients had pancreatic and prostate cancer in each dose and duration categories and the confidence intervals were wide (Supplementary Table S4).

### Analysis using age as timescale in the Cox model

Results were similar between the analysis using age as the timescale and that using time since starting insulin in the Cox model (Table 7). A significantly higher risk associated with insulin glargine use was also found for pancreatic cancer, particularly in men, and for prostate cancer.

**Table 7:** Hazard ratio of overall and individual cancer comparing exclusive users of insulin glargine vs. intermediate/long-acting human insulin (HI) using age as timescale in the Cox proportional hazard model.

	Using age as timescale, unadjusted	Using age as timescale and stratified on birth cohort, unadjusted	Using age as time scale and stratified on birth cohort, adjusted for baseline propensity score
Any cancer	0.91 (0.78, 1.06)	0.91 (0.78, 1.06)	0.86 (0.72, 1.02)
Breast (women)	0.68 (0.29, 1.57)	0.65 (0.28, 1.51)	0.56 (0.23, 1.37)
Colorectal	0.85 (0.55, 1.31)	0.86 (0.56, 1.33)	0.86 (0.54, 1.38)
Stomach	0.70 (0.32, 1.52)	0.71 (0.33, 1.53)	0.63 (0.28, 1.44)
Pancreas	2.31 (1.44, 3.69)	2.26 (1.41, 3.63)	1.81 (1.05, 3.11)
Pancreas (men)	2.13 (1.11, 4.08)	2.07 (1.07, 3.98)	2.20 (1.04, 4.69)
Liver	0.74 (0.53, 1.01)	0.73 (0.53, 1.01)	0.72 (0.51, 1.01)
Lung	1.08 (0.67, 1.74)	1.07 (0.66, 1.73)	0.98 (0.58, 1.66)
Prostate (men)	2.28 (1.08, 4.83)	2.39 (1.12, 5.09)	2.59 (1.04, 6.45)
Kidney and urinary bladder	0.64 (0.31, 1.31)	0.63 (0.31, 1.29)	0.56 (0.26, 1.21)
Skin	1.17 (0.56, 2.46)	1.17 (0.56, 2.47)	1.15 (0.51, 2.58)

## Discussion

In a Chinese population with high incidence of colorectal, liver, and lung cancers, insulin glargine was not associated with an excess risk of overall cancer as compared to intermediate/long-acting HI among adult type 2 diabetes patients over an average follow-up of 2 years. This result is consistent with the finding from a randomized controlled trial enrolling 1,017 type 2 diabetes patients comparing insulin glargine with neutral protamine Hagedorn insulin [4]. In that study patients were followed for 5 years and no difference in cancer risk was observed. However, a potentially increased risk of pancreatic and prostate cancer was found in our study which required further investigations for confirmation.

Evidence suggests a direct link between serum insulin and elevated blood glucose levels and several cancer sites [13], [14]. Therefore, confounding by underlying diabetes severity or other risk factors for cancer deserves special attention [11]. The incomparability between insulin glargine users and other types of insulin or oral anti-diabetic agent users raises the question about the most appropriate reference group in the studies of cancer risk and insulin. In Taiwan, insulin glargine has been recommended to be used among patients with poor controlled diabetes or frequent episodes of hypoglycemia. In this study intermediate/long-acting HI was selected as the comparison group because both were used as alternatives for basal insulin supplement. However, there were still significant differences in the baseline characteristics, including comorbidities, diabetic complications, and other medications between the two groups. Our analyses adjusted for these baseline imbalances between insulin glargine and intermediate/long-acting HI initiators, but we could not rule out the possibility of residual confounding.

Additionally, insulin glargine group had a higher mean daily dosage of sulfonylurea and metformin concomitantly after treatment initiation. In contrast to a German study that an excess cancer risk with insulin glargine emerged after adjustment for dose [6], our risk estimates for overall cancer and individual sites of cancer did not change substantially after adjusting for insulin dosage. In this study, we used an extended Cox model to adjust for time-varying covariates. Other more sophisticated approaches, including marginal structural model, might provide less biased estimates [15].

Other potential bias that may explain an association between insulin glargine use and cancer include reverse causation – cancer may affect glycemic control (manifest as either hyper- or hypoglycemia) that leads physicians to switch to insulin glargine [11]. Analysis including only “exclusive users”, although reduce the biases from exposure misclassification which may bias effects toward the null, on the other hand, may be open to bias due to informative censoring [16]. Therefore, we conducted additional analysis using a “new-user design” that required patients who did not have a history of cancer to be free of any studied insulin use during the 6-month period preceding the start of insulin treatment and censored patients when they stopped using glargine insulin or intermediate/long-acting HI or started using another study insulin [17]. Despite this design allowed us to estimate the incidence of cancer following a new episode of insulin treatment, follow-up duration was substantially shorter as compared with analysis on exclusive users which led to imprecise risk estimates. A nested case-control design comparing cumulative exposure of insulin glargine and intermediate/long-acting HI between cancer cases and time-matched control may analyze the data more efficiently.

Despite no excess of overall cancer incidence, our study showed that insulin glargine might be associated with a higher chance of pancreatic cancer and prostate cancer diagnosis. Due to few number of cancer cases, we could not evaluate the potential dose and duration of insulin glargine use that increased the occurrence of these two cancers. The mechanisms leading to this positive association was uncertain, as pancreatic cancer cells were previously shown to respond similarly to insulin glargine and human insulin, and survival in insulin glargine-treated patients after treatment for pancreatic cancer was similar to those on human insulin [18]. However, due to the possibility that insulin analogues may promote the growth of subclinical tumors in a relative short duration of exposure, this potential risk deserved attention and needed to be evaluated in further studies. Similar to prior reports, the duration of exposure to insulin glargine in the present study was less than what would be reasonably anticipated for establishing a causal relationship with carcinogenicity. Taken together, these observations suggest that the use of insulin glargine increases the rate of development and subsequent detection of pre-existing undetectable malignancies rather than malignant cell transformation and new cancer formation. In contrast to a significantly increased risk of breast cancer associated with glargine use reported by the Swedish and the Scottish studies [8], [9], our study did not find a relation with breast cancer.

Our study has several limitations. First, we were not able to examine the long-term effect of insulin glargine on cancer although our average length of follow-up is comparable or slightly longer than previous studies. Second, as discussed above, there might be residual confounding by duration or severity of diabetes, as well as by obesity, smoking, and physical inactivity. Due to lack of data about the exact extent of glycemic control, we could not examine whether hyperglycemia *per se* or a higher dose of insulin glargine attributes to the association with a greater risk of certain specific cancer. Third, more frequent clinical visits and hospitalizations among insulin glargine users might have led to more cancer cases being detected in these patients. However, if there was a detection bias, we would expect to see a similarly increased risk for individual sites cancers, including breast, colorectal and liver cancer. And finally, we could not exclude the possibility that some of the associations might be due to chance, as multiple individual sites of cancer were examined simultaneously.

In conclusion, we did not find an increased risk of overall cancer incidence associated with insulin glargine use among type 2 diabetes patients. The positive associations between insulin glargine and pancreatic and prostate cancer definitely require further investigations.

## Materials and Methods

### Ethics

The National Taiwan University Hospital Research Ethics Committee approved the protocol of this study and waived the need for written informed consent because this is a retrospective study based on data from administrative databases and involved only minimal risk.

### Data Source

The Taiwan National Health Insurance database includes complete outpatient visits, hospital admissions, diagnoses, prescriptions, disease and vital status for 99% of the population (about 23 million) in Taiwan. We established a longitudinal medical history of each beneficiary by linking the computerized administrative and claims datasets, and the National Cancer and Death Registry through the civil identification number unique to each beneficiary and date of birth. Our source population comprised all beneficiaries aged ≥18 years between January 1, 2004 and December 31, 2007.

### Study Population

From the source population, we identified beneficiaries with a first prescription of either insulin glargine (anatomical therapeutic chemical [ATC] classification system code A10AE04) or intermediate/long-acting HI (ATC codes A10AC01 and A10AE01) between July 1, 2004 and June 30, 2007 (insulin glargine entered Taiwan's market in 2004). We refer to the date of first prescription of either insulin therapy as the index date. We required eligible beneficiaries to have no prescription of either insulin glargine or intermediate/long-acting HI in the 6 months preceding the index date. Intermediate/long-acting HI was used as the active comparator for insulin glargine because both were used as alternatives for basal insulin supplement and we expected the characteristics between two treatment groups to be more similar as compared with other types of insulin or analogues.

To exclude patients with potential type 1 diabetes, we excluded those who 1) had a hospital admission with a discharge diagnosis of insulin dependent diabetes mellitus (International Classification of Diseases, 9^th^ Revision, Clinical Modification [ICD-9-CM] codes 250.x1, 250.x3), or 2) received a catastrophic illness certificate issued by the Department of Health for type 1 diabetes. We further excluded patients who 1) did not have continuous insurance coverage 6 months preceding the index date, 2) had a history of cancer recorded in the National Cancer Registry anytime before the index date, and 3) received both insulin glargine and intermediate/long-acting HI on the index date.

The primary outcome of this study was any cancer, and the secondary outcomes were specific site of cancer, including liver, colorectal, pancreatic, lung, kidney or urinary bladder, stomach, skin, breast, and prostate cancer. All of the cancer occurrences were identified and validated by linkage through the National Cancer Registry. National Cancer Registry in Taiwan was launched in 1979 to collect information of all incident cancer cases from hospitals with 50 or more beds. It was considered a complete and accurate registry with a percentage of cases based on death certificate only as low as 3.9% in 2000 and 1.8% in 2005.

### Covariate Ascertainment and Adjustment

We used inpatient and outpatient diagnosis files and prescription file during the 6-month period before the index date to ascertain patients' history of cardiovascular, peripheral vascular, cerebrovascular disease, metabolic derangement (diabetes with ketoacidosis, hyperosmolarity, and with other coma), retinopathy, nephropathy, neuropathy, and depression (ICD-9-CM codes provided in Supplementary Table S1); and use of biguanides (A10BA), sulfonylurea (A10BB), alpha-glucosidase inhibitors (A10BF), thiazolidinediones (A10BG), glinides (A10BX02, A10BX03), detemir insulin (A10AE05), fast-acting insulins and analogues (A10AB), low-dose aspirin (B01AC06), and statins (C10AA). Using these covariates plus age (in five categories), sex, studied insulin initiation year (1-year band), physicians' characteristics and patients' health services utilization (number of outpatient visits, number of hospitalizations, use of preventive medicine services) in the 6 months preceding the index date, we estimated the propensity score – the probability of initiating insulin glargine – with a logistic regression model.

For use of other types of insulin and oral anti-diabetic agents, we further identified date of prescription, days supplied, and total amount prescribed. For each patient we estimated mean daily dose by calculating cumulative dose prescribed divided by the total follow-up duration at risk. Data were presented as the number of defined daily doses (DDD) which was established by an expert panel as the typical maintenance dose required when the drug is used for its main indication in an adult [19].

### Statistical Analysis

Baseline characteristics, comorbidities, medication use, and health services utilization related to starting insulin glargine rather than intermediate/long-acting HI therapy were identified by calculating odds ratios and their 95% CIs from a Logistic regression model. We estimated the incidence rate and its 95% confidence interval (CI) of overall cancer and specific site of cancer based on a Poisson distribution.

In the “exclusive users” analysis, we restricted to patients who used insulin glargine or HI but not both during the whole study period and following them to the earliest of cancer diagnosis, death, disenrollment, or December 31 2007. We estimated the hazard ratio and its 95% CI of all cancer and individual site of cancer comparing insulin glargine initiators with intermediate/long-acting HI initiators by fitting five different Cox proportional hazards models: 1) unadjusted, 2) adjusted for all baseline variables in Table 1 by a traditional multivariable model, 3) adjusted for baseline propensity score in deciles, 4) adjusted for baseline propensity score and time-varying use of insulin detemir, mean daily dosage of fast-acting insulin, sulfonylurea, and metformin use in quartiles after the index date, and 5) adjusted for baseline propensity score, time-varying use of insulin detemir, mean daily dosage of fast-acting insulin, sulfonylurea, and metformin use in quartiles, and time-varying mean daily dosage of insulin glargine or intermediate/long-acting HI (≥0.5 DDD/day and <0.5 DDD/day). These hypoglycemic agents and insulin glargine dosage have been reported to be associated with cancer risks and could potentially confound the risk estimates between two treatment groups. Analyses adjusted for baseline propensity score were further stratified by men and women to see if there was any treatment by gender interaction. We tested the proportional hazards assumption by including a treatment-by-time interaction term.

We also conducted additional analyses that 1) followed all studied insulin users based on their treatment status on the index date to the earliest of cancer diagnosis, death, disenrollment, discontinuing studied insulin or switching to another insulin, or study end (“as-treated analysis”), and 2) using age as the timescale (i.e. with the same entry and exit dates for each person but declaring date of birth as the origin) in the Cox model while stratifying by birth cohort (10-year intervals) for a better control of age and calendar effects (“age as the timescale”). In addition, we classified exposure person-days of studied insulin use into cumulative dose (in quartiles); cumulative duration ≥1 and <1 year; and mean daily dosage ≥0.5 DDD/day and <0.5DDD/day; and calculated the hazard ratios of insulin glargine use vs. HI use for each category. Potential dose and duration response were explored when an association with specific cancer site was found.

All statistical analyses were performed with SAS 9.2 (SAS Institute, Cary, NC).

## Supporting Information

Table S1 ICD-9-CM diagnostic codes used to identify patients with comorbid diseases.
(DOC)

Table S2 Hazard ratio of overall and individual cancer comparing insulin glargine with intermediate/long-acting human insulin (HI) users by as-treated analysis.
(DOC)

Table S3 Hazard ratio of overall and individual cancer comparing insulin glargine with intermediate/long-acting human insulin (HI) among men and women by as-treated analysis.
(DOC)

Table S4 Hazard ratio of overall cancer, pancreatic and prostate cancer associated with insulin glargine, compared with intermediate/long-acting human insulin (HI) by as-treated analysis.
(DOC)
