# Supplementary material for: Cancer Risk Associated with Insulin Glargine among Adult Type 2 Diabetes Patients – A Nationwide Cohort Study
Source: PLoS One. 2011 Jun 27;6(6):e21368. doi: 10.1371/journal.pone.0021368 (PMC3124499; doi:10.1371/journal.pone.0021368)
Supplement: Table S1 — ICD-9-CM diagnostic codes used to identify patients with comorbid diseases. (DOC) [file pone.0021368.s001.doc]

**Supplementary Table 1** ICD-9-CM diagnostic codes used to identify patients with comorbid diseases

| Comorbidities | ICD-9-CM codes |
| --- | --- |
| Cardiovascular disease | 250.7, 401-405, 410-414, 425-428, 429.1-429.3, 441, 442, 458 |
| Ischemic heart disease | 410-414 |
| Peripheral vascular disease | 441, 443.9, 785.4, V43.4, 38.48(Procedure) |
| Cerebrovascular disease | 430-438 |
| Ketoacidosis or hyperosmolarity | 250.1, 250,2 |
| Retinopathy | 250.5, 362, 364.0, 364.4, 365-366, 368-369, 377 |
| Neuropathy | 250.6, 337.1, 354, 355, 356.8, 357.2, 358.1, 713.5, 729.2 |
| Nephropathy | 250.4, 580-588, 590, 593, 595, 596, 599, 791.0 |
| Chronic kidney disease | 403.01, 403.11, 403.91, 404.02, 404.03, 404.12, 404.13, 404.92, 404.93, 585, V45.1, V56.0, V56.8 |
| Chronic liver disease | 571, 070.41, 070.44, 070.51, 070.54, V02.62, 070.22, 070.23, 070.33, V02.61, 291.xx, 303.0x, 303.9x, 305.0x |
| Chronic lung disease | 490-496, 500-508 |
| Major depression | 300.4, 311, V79.0,296.2, 296.3, 296.5, 296.82 |
