# Supplementary material for: Cancer Risk Associated with Insulin Glargine among Adult Type 2 Diabetes Patients – A Nationwide Cohort Study
Source: PLoS One. 2011 Jun 27;6(6):e21368. doi: 10.1371/journal.pone.0021368 (PMC3124499; doi:10.1371/journal.pone.0021368)
Supplement: Table S2 — Hazard ratio of overall and individual cancer comparing insulin glargine with intermediate/long-acting human insulin (HI) users by as-treated analysis. (DOC) [file pone.0021368.s002.doc]

**Supplementary Table 2** Hazard ratio of overall and individual cancer comparing insulin glargine with intermediate/long-acting human insulin (HI) users by as-treated analysis

|  | Unadjusted | Traditional multivariable adjusted ^a^ | Adjusted for baseline propensity score | Adjusted for baseline propensity score and time-varying medication use ^b^ | Adjusted for baseline propensity score, time-varying medication use and dosage of studied insulin |
| --- | --- | --- | --- | --- | --- |
| Any cancer | 0.83  (0.65, 1.06) | 0.87  (0.75, 1.02) | 0.81  (0.62, 1.05) | 0.85  (0.65, 1.13) | 0.86  (0.65, 1.13) |
| Breast (women) | 0.52  (0.12, 2.28) | 0.51  (0.11, 2.51) | 0.53  (0.11, 2.55) | 0.40  (0.08, 1.98) | 0.40  (0.08, 1.97) |
| Colorectal | 0.93  (0.48, 1.80) | 1.50  (0.73, 3.08) | 1.09  (0.53, 2.55) | 1.14  (0.54, 2.38) | 1.15  (0.55, 2.41) |
| Stomach | 0.38  (0.09, 1.64) | 0.75  (0.16, 3.61) | 0.47  (0.10, 2.31) | 0.48  (0.10, 2.35) | 0.48  (0.10, 2.34) |
| Pancreas | 1.97  (0.96, 4.07) | 2.11  (0.93, 4.78) | 1.76  (0.77, 4.03) | 1.81  (0.77, 4.27) | 1.80  (0.76, 4.26) |
| Liver | 0.78  (0.48, 1.26) | 1.03  (0.61, 1.73) | 0.74  (0.44, 1.25) | 0.82  (0.48, 1.41) | 0.81  (0.47, 1.40) |
| Lung | 0.68  (0.29, 1.62) | 0.81  (0.32, 2.05) | 0.57  (0.23, 1.44) | 0.74  (0.29, 1.91) | 0.75  (0.29, 1.93) |
| Prostate (men) | 3.75  (1.24, 11.32) | 7.21  (1.78, 29.21) | 4.44  (1.12, 17.68) | 6.35  (1.34, 30.02) | 6.46  (1.37, 30.51) |
| Kidney and bladder | 0.61  (0.24, 1.58) | 1.09  (0.39, 3.07) | 0.84  (0.31, 2.31) | 0.87  (0.31, 2.46) | 0.87  (0.31, 2.45) |
| Skin | 0.65  (0.19, 2.18) | 1.15  (0.31, 4.24) | 0.80  (0.21, 3.01) | 0.82  (0.21, 3.25) | 0.83  (0.21, 3.26) |

^a^ Adjusted for all variables in Table 1

^b^ Time-varying medication use included insulin detemir (binary), mean daily dosage of fast-acting insulins, sulfonylurea, and metformin (in quartiles)
