# Supplementary material for: Cancer Risk Associated with Insulin Glargine among Adult Type 2 Diabetes Patients – A Nationwide Cohort Study
Source: PLoS One. 2011 Jun 27;6(6):e21368. doi: 10.1371/journal.pone.0021368 (PMC3124499; doi:10.1371/journal.pone.0021368)
Supplement: Table S3 — Hazard ratio of overall and individual cancer comparing insulin glargine with intermediate/long-acting human insulin (HI) among men and women by as-treated analysis. (DOC) [file pone.0021368.s003.doc]

**Supplementary Table 3** Hazard ratio of overall and individual cancer comparing insulin glargine with intermediate/long-acting human insulin (HI) among men and women by as-treated analysis

|  | Women | | Men | |
| --- | --- | --- | --- | --- |
|  | Unadjusted | Adjusted for baseline propensity score | Unadjusted | Adjusted for baseline propensity score |
| Any cancer | 0.79  (0.62, 0.99) | 0.76  (0.49, 1.17) | 0.91  (0.75, 1.10) | 0.83  (0.59, 1.17) |
| Colorectal | 1.94  (0.77, 4.90) | 1.51  (0.53, 4.26) | 0.92  (0.35, 2.38) | 0.80  (0.28, 2.27) |
| Stomach | 0.63  (0.08, 4.97) | 0.25  (0.03, 2.23) | 0.44  (0.06, 3.42) | 1.25  (0.15, 10.82) |
| Pancreas | 1.03  (0.23, 4.67) | 0.53  (0.11, 2.61) | 3.86  (1.61, 9.24) | 3.38  (1.18, 9.66) |
| Liver | 0.96  (0.40, 2.28) | 0.80  (0.31, 2.06) | 1.11  (0.62, 1.97) | 0.70  (0.37, 1.32) |
| Lung | 0.55  (0.07, 4.34) | 0.28  (0.03, 2.40) | 1.06  (0.41, 2.77) | 0.71  (0.26, 1.98) |
| Kidney and urinary bladder | 1.27  (0.36, 4.46) | 1.46  (0.36, 5.95) | 0.55  (0.13, 2.39) | 0.51  (0.11, 2.33) |
| Skin | 0.44  (0.06, 3.38) | 0.38  (0.05, 3.28) | 1.90  (0.38, 9.50) | 1.75  (0.29, 10.63) |
