# Supplementary material for: Cancer Risk Associated with Insulin Glargine among Adult Type 2 Diabetes Patients – A Nationwide Cohort Study
Source: PLoS One. 2011 Jun 27;6(6):e21368. doi: 10.1371/journal.pone.0021368 (PMC3124499; doi:10.1371/journal.pone.0021368)
Supplement: Table S4 — Hazard ratio of overall cancer, pancreatic and prostate cancer associated with insulin glargine, compared with intermediate/long-acting human insulin (HI) by as-treated analysis. (DOC) [file pone.0021368.s004.doc]

**Supplementary Table 4** Hazard ratio of overall cancer, pancreatic and prostate cancer associated with insulin glargine, compared with intermediate/long-acting human insulin (HI) by as-treated analysis

|  | Hazard Ratio (95% CI) | | |
| --- | --- | --- | --- |
|  | Crude | Adjusted for baseline propensity score ^a^ | Adjusted for baseline propensity score and time-varying medication use ^b^ |
| Overall cancer |  |  |  |
| Cumulative dosage |  |  |  |
| ≥180 DDD | 0.83 (0.56, 1.24) | 0.89 (0.57, 1.40) | 0.97 (0.62, 1.54) |
| <180 DDD | 0.82 (0.60, 1.12) | 0.73 (0.52, 1.03) | 0.73 (0.52, 1.03) |
| Cumulative duration |  |  |  |
| ≥1 years | 0.86 (0.51, 1.43) | 0.89 (0.51, 1.58) | 0.97 (0.54, 1.74) |
| <1 years | 0.81 (0.61, 1.07) | 0.75 (0.55, 1.03) | 0.77 (0.56, 1.05) |
| Mean daily dosage |  |  |  |
| ≥0.5 DDD/day | 0.76 (0.57, 1.01) | 0.76 (0.56, 1.03) | 0.78 (0.57, 1.07) |
| <0.5 DDD/day | 1.10 (0.65, 1.85) | 0.95 (0.53, 1.69) | 0.92 (0.51, 1.65) |
| Pancreatic cancer |  |  |  |
| Cumulative dosage |  |  |  |
| ≥180 DDD | 0.54 (0.07, 4.28) | 0.53 (0.06, 4.69) | 0.55 (0.06, 5.17) |
| <180 DDD | 2.86 (1.25, 6.53) | 2.51 (0.94, 6.69) | 2.37 (0.87, 6.41) |
| Cumulative duration |  |  |  |
| ≥1 years | 1.32 (0.15, 11.63) | 1.76 (0.17, 18.88) | 2.40 (0.21, 27.44) |
| <1 years | 2.09 (0.96, 4.53) | 1.76 (0.71, 4.35) | 1.70 (0.68, 4.26) |
| Mean daily dosage |  |  |  |
| ≥0.5 DDD/day | 3.12 (0.62, 15.72) | 2.49 (0.43, 14.38) | 2.55 (0.41, 15.97) |
| <0.5 DDD/day | 1.74 (0.76, 3.97) | 1.60 (0.61, 4.21) | 1.51 (0.56, 4.08) |
| Prostate cancer (men) |  |  |  |
| Cumulative dosage |  |  |  |
| ≥180 DDD | 2.56 (0.63, 10.35) | 2.05 (0.39, 10.79) | 2.74 (0.48, 15.64) |
| <180 DDD | 9.98 (1.03, 96.97) | 30.34 (2.05, 448.25) | 27.89 (1.98, 392.35) |
| Cumulative duration |  |  |  |
| ≥1 years | 2.19 (0.42, 11.42) | 1.74 (0.27, 11.32) | 2.06 (0.29, 14.78) |
| <1 years | 6.71 (1.23, 36.79) | 15.52 (1.79, 134.45) | 17.93 (2.06, 155.93) |
| Mean daily dosage |  |  |  |
| ≥0.5 DDD/day | 3.79 (1.26, 11.40) | 4.22 (1.02, 17.43) | 5.33 (1.20, 23.73) |
| <0.5 DDD/day | NA | NA | NA |

^a^ Extended Cox model with time-varying insulin glargine and HI use, controlling for baseline propensity score

^b^ Extended Cox model with time-varying insulin glargine and HI use, controlling for baseline propensity score and time-varying use of insulin detemir (binary), mean daily dosage of fast-acting insulins, sulfonylurea, and metformin (in quartile)

NA: Not available
